# Supplementary material for: Unraveling the Origin of Social Bursts in Collective Attention
Source: Sci Rep. 2020 Mar 13;10:4629. doi: 10.1038/s41598-020-61523-z (PMC7069943; doi:10.1038/s41598-020-61523-z)
Supplement: Supplementary file 1 — Supplementary Information. [file 41598_2020_61523_MOESM1_ESM.pdf]

Unraveling the Origin of Social Bursts in Collective Attention

Manlio De Domenico[12*] **and Eduardo G. Altmann**[23]

[1]CoMuNe Lab, Fondazione Bruno Kessler, Via Sommarive 18, 38123 Povo (TN), Italy.
[2]Max Planck Institute for the Physics of Complex Systems, 01187 Dresden, Germany
[3]School of Mathematics and Statistics, The University of Sydney, 2006 NSW, Australia
[*]To whom correspondence should be addressed; E-mail: mdedomenico@fbk.eu.

ABSTRACT

In the era of social media, every day billions of individuals produce content in socio-technical systems resulting in a deluge of information. However, human attention is a limited resource and it is increasingly challenging to consume the most suitable content for one's interests. In fact, the complex interplay between individual and social activities in social systems overwhelmed by information results in bursty activity of collective attention which are still poorly understood. Here, we tackle this challenge by analyzing the online activity of millions of users in a popular microblogging platform during exceptional events, from NBA Finals to the elections of Pope Francis and the discovery of gravitational waves. We observe extreme fluctuations in collective attention that we are able to characterize and explain by considering the co-occurrence of two fundamental factors: the heterogeneity of social interactions and the preferential attention towards influential users. Our findings demonstrate how combining simple mechanisms provides a route towards complex social phenomena.

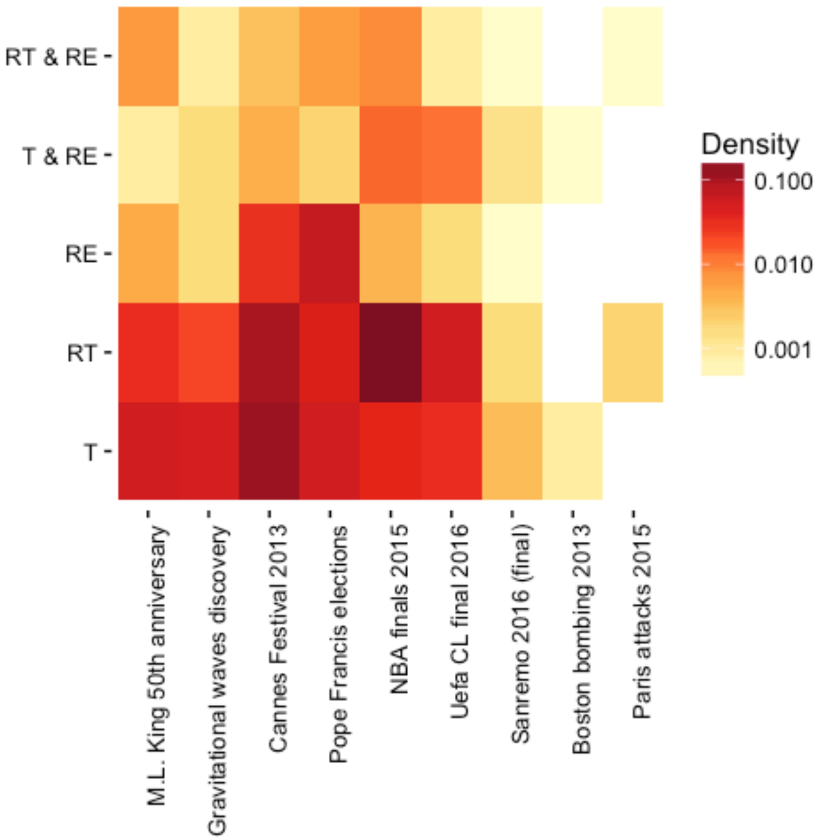

Supplementary Figure 1. Density of social bursts. Fraction of bursty activity due to specific actions (T = tweet, RT = Retweet, RE = Reply) and their combinations during 9 exceptional events (see Main Text for an overview of the data sets).

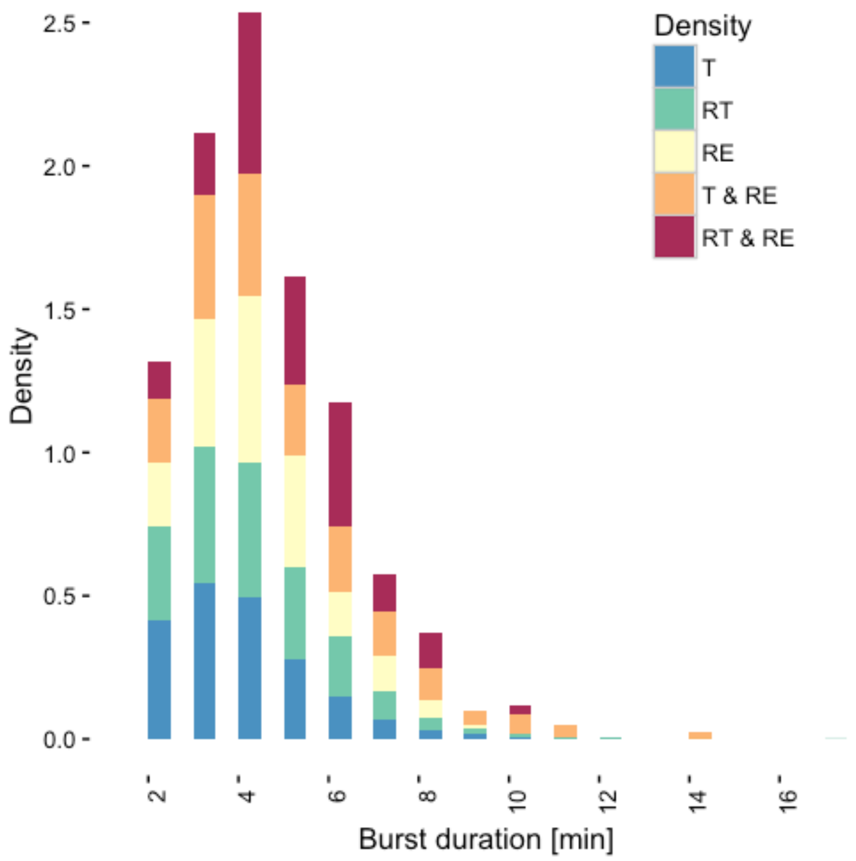

Supplementary Figure 2. Duration of social bursts. Distribution of bursts' duration due to specific actions (T = tweet, RT = Retweet, RE = Reply) and their combinations measured from all the exceptional events considered in this study (see Main Text for an overview of the data sets).

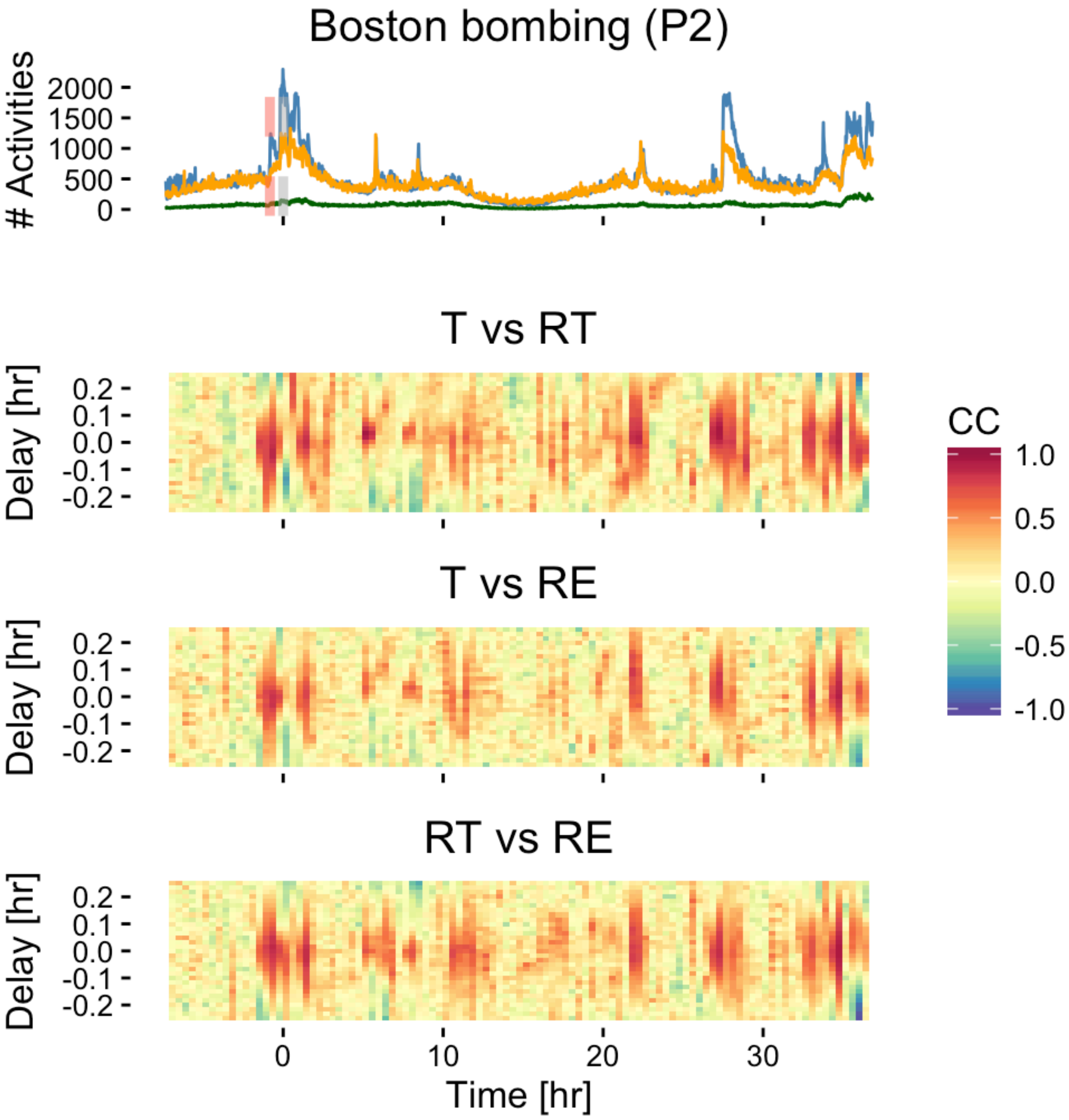

Supplementary Figure 3. Correlated activity during collective attention. From top to bottom: i) volume per minute of non-social (orange) and social activities (light blue for retweets, dark green for replies); ii) cross-correlation, color coded, as a function of temporal delay (*y* axis) and natural time (*x* axis) between different activities.

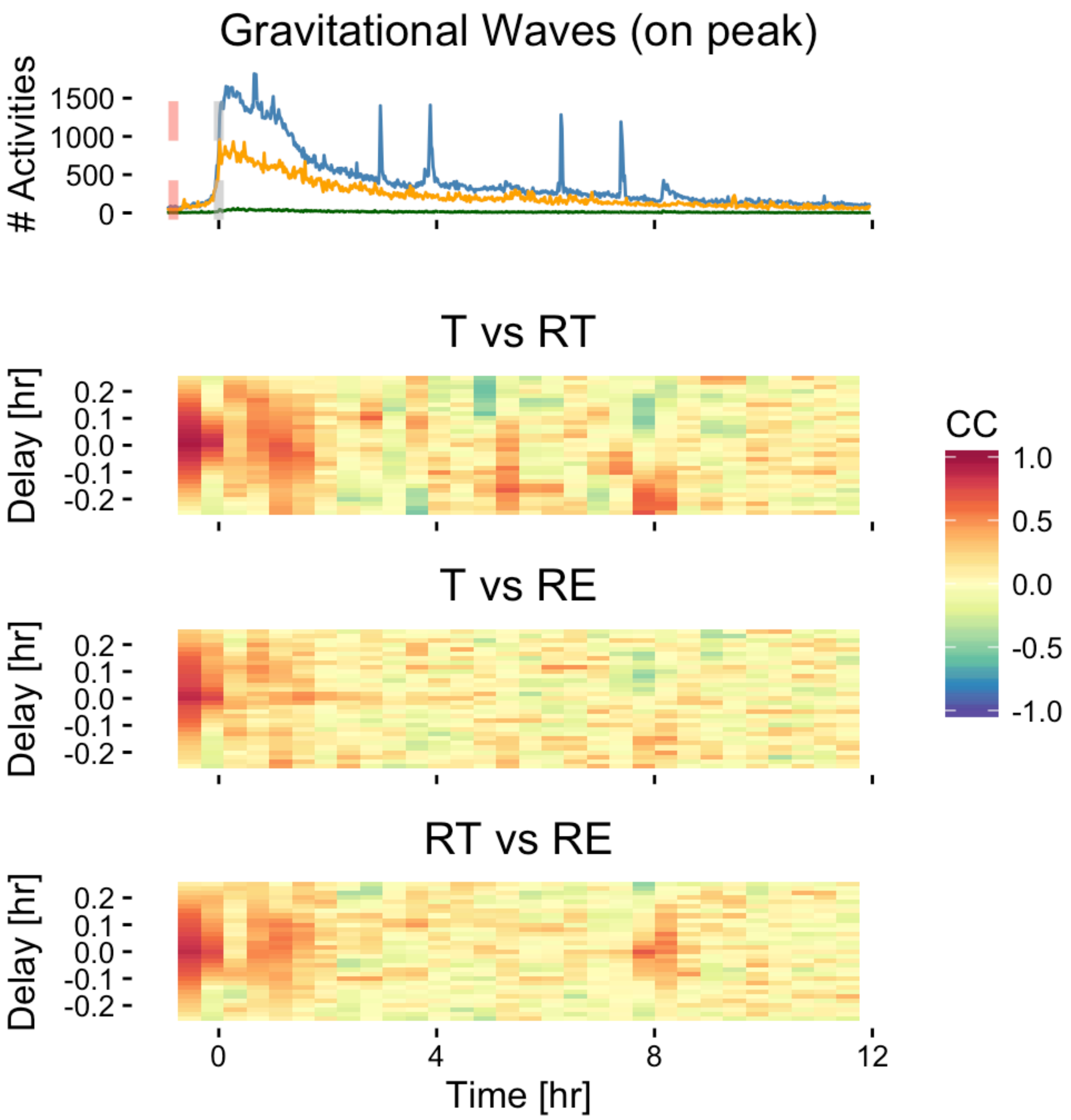

Supplementary Figure 4. Correlated activity during collective attention. From top to bottom: i) volume per minute of non-social (orange) and social activities (light blue for retweets, dark green for replies); ii) cross-correlation, color coded, as a function of temporal delay (*y* axis) and natural time (*x* axis) between different activities.

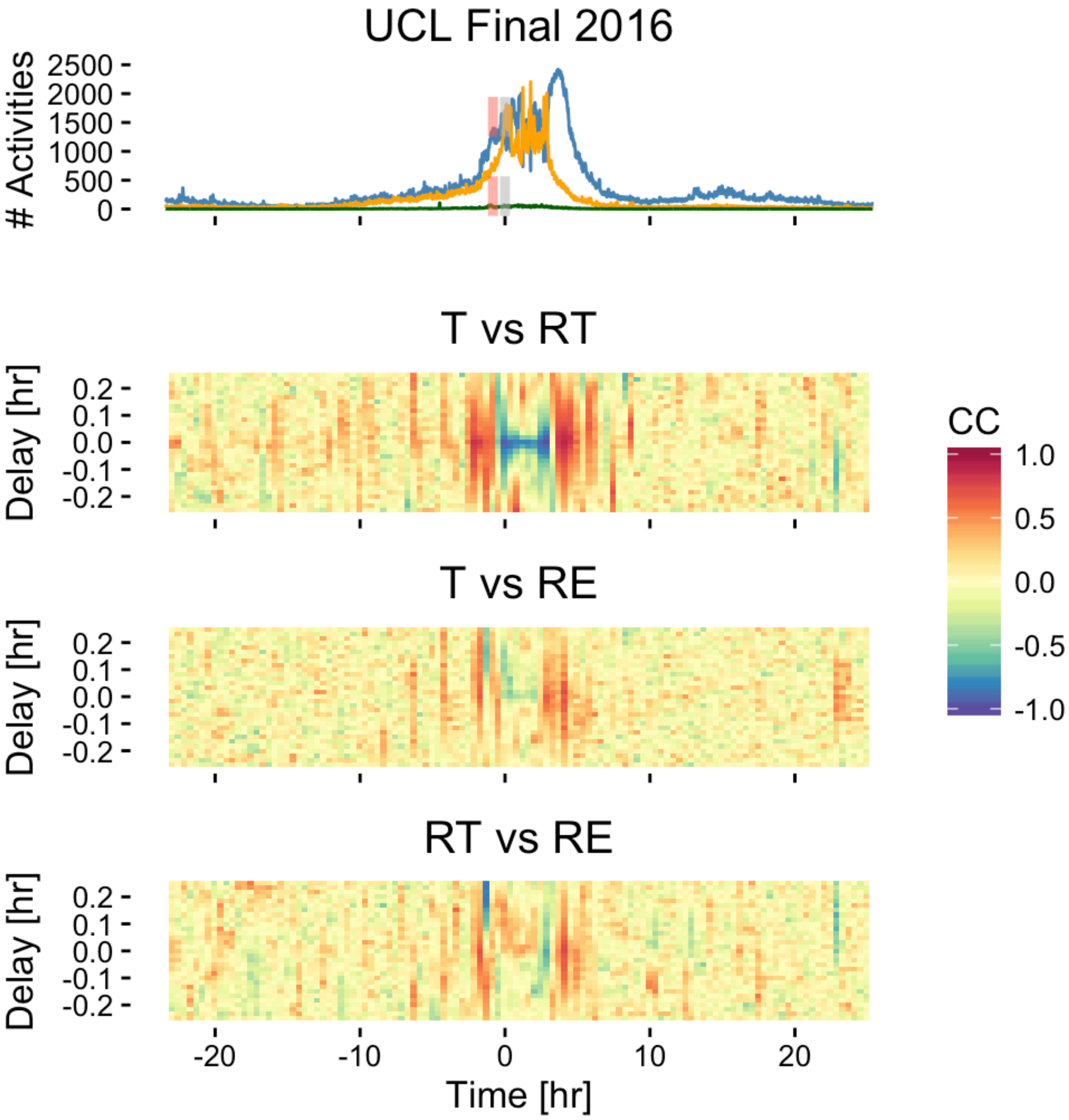

Supplementary Figure 5. Correlated activity during collective attention. From top to bottom: i) volume per minute of non-social (orange) and social activities (light blue for retweets, dark green for replies); ii) cross-correlation, color coded, as a function of temporal delay (*y* axis) and natural time (*x* axis) between different activities.

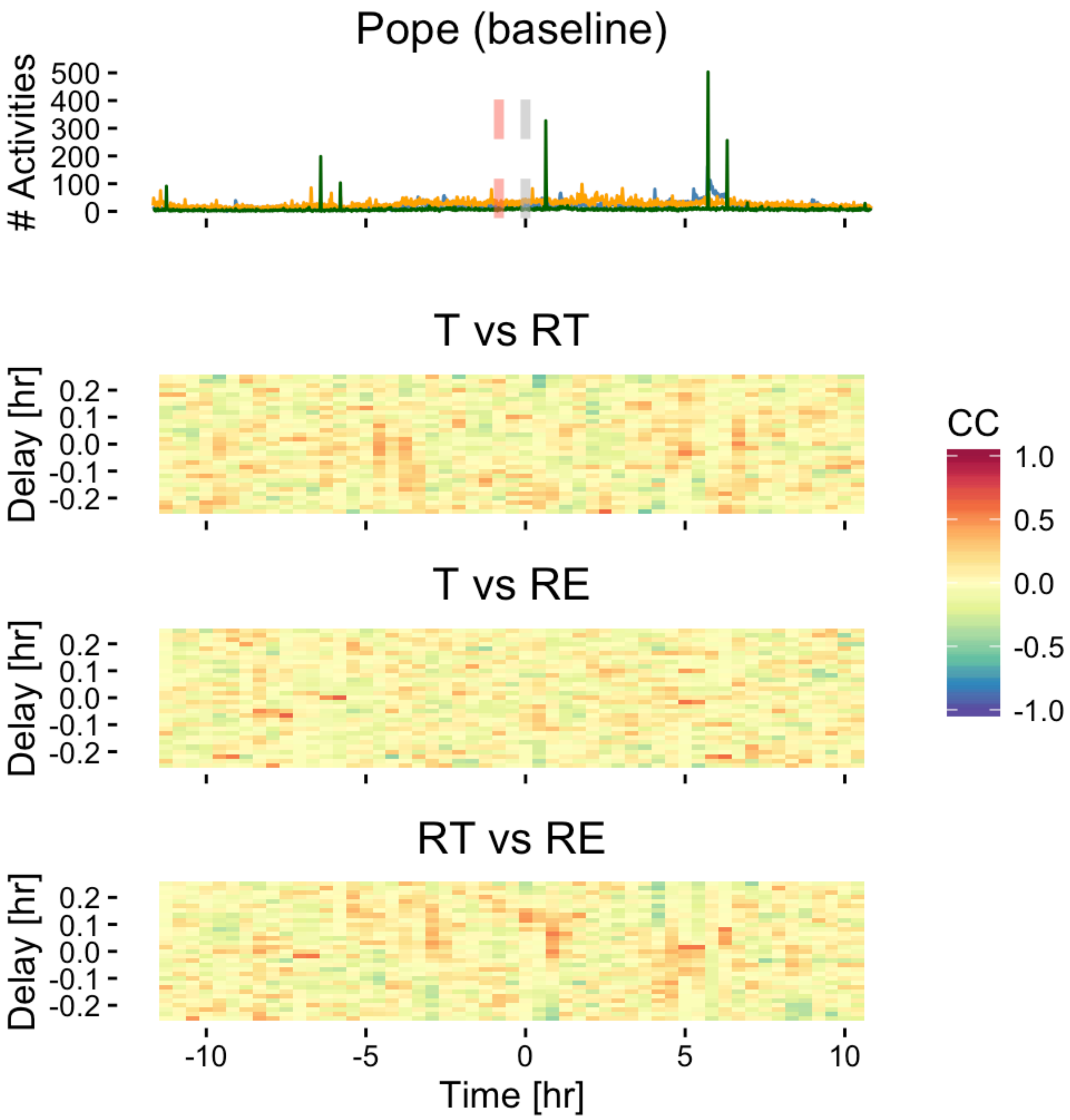

Supplementary Figure 6. Correlated activity during collective attention. From top to bottom: i) volume per minute of non-social (orange) and social activities (light blue for retweets, dark green for replies); ii) cross-correlation, color coded, as a function of temporal delay (*y* axis) and natural time (*x* axis) between different activities.

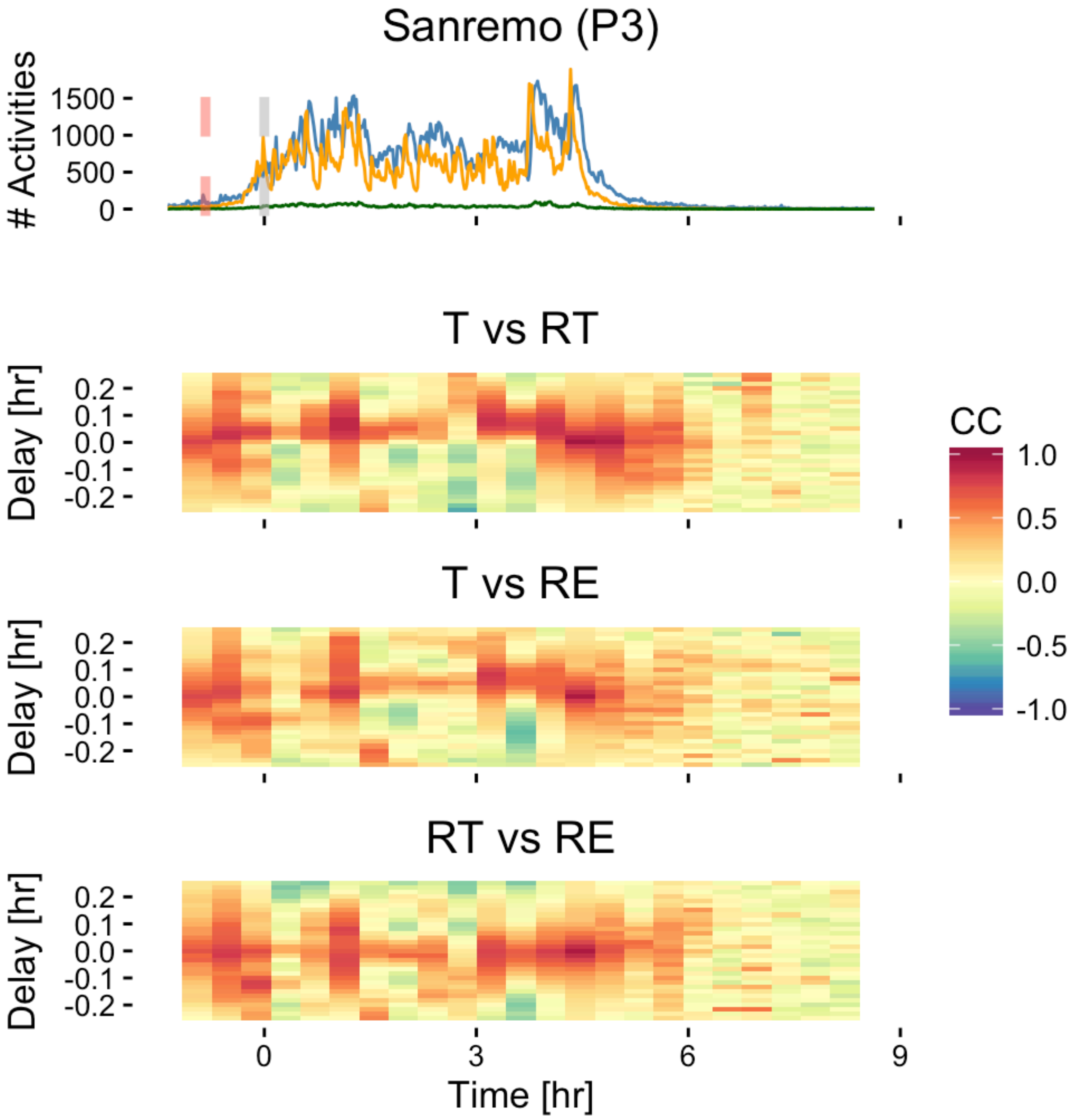

Supplementary Figure 7. **Correlated activity during collective attention.** From top to bottom: i) volume per minute of non-social (orange) and social activities (light blue for retweets, dark green for replies); ii) cross-correlation, color coded, as a function of temporal delay (*y* axis) and natural time (*x* axis) between different activities.
